# Noise reduction by adaptive-SIN filtering for retinal OCT images

**DOI:** 10.1038/s41598-021-98832-w

**Published:** 2021-09-30

**Authors:** Yan Hu, Jianfeng Ren, Jianlong Yang, Ruibing Bai, Jiang Liu

**Affiliations:** 1 Department of Computer Science and Engineering, Southern University of Science and Technology, Shenzhen, 518055 China; 2 Departement of Computer Science, Faculty of Science and Engineering, University of Nottingham, Ningbo, China; 3 School of Biomedical Engineering, Shanghai Jiao Tong University, Shanghai, China; 4 Guangdong Provincial Key Laboratory of Brain-Inspired Intelligent Computation, Department of Computer Science and Engineering, Southern University of Science and Technology, Shenzhen, 518055 China

**Keywords:** Biomedical engineering, Tomography

## Abstract

Optical coherence tomography (OCT) images is widely used in ophthalmic examination, but their qualities are often affected by noises. Shearlet transform has shown its effectiveness in removing image noises because of its edge-preserving property and directional sensitivity. In the paper, we propose an adaptive denoising algorithm for OCT images. The OCT noise is closer to the Poisson distribution than the Gaussian distribution, and shearlet transform assumes additive white Gaussian noise. We hence propose a square-root transform to redistribute the OCT noise. Different manufacturers and differences between imaging objects may influence the observed noise characteristics, which make predefined thresholding scheme ineffective. We propose an adaptive 3D shearlet image filter with noise-redistribution (adaptive-SIN) scheme for OCT images. The proposed adaptive-SIN is evaluated on three benchmark datasets using quantitative evaluation metrics and subjective visual inspection. Compared with other algorithms, the proposed algorithm better removes noise in OCT images and better preserves image details, significantly outperforming in terms of both quantitative evaluation and visual inspection. The proposed algorithm effectively transforms the Poisson noise to Gaussian noise so that the subsequent shearlet transform could optimally remove the noise. The proposed adaptive thresholding scheme optimally adapts to various noise conditions and hence better remove the noise. The comparison experimental results on three benchmark datasets against 8 compared algorithms demonstrate the effectiveness of the proposed approach in removing OCT noise.

## Introduction

Optical Coherence Tomography (OCT)^1^ is an established medical imaging technique that captures micrometer-resolution, three-dimensional images by imposing light on optical scattering media such as biological tissue. The resolutions are in the range of 1 to 15 µm, smaller than that of ultrasound imaging^2^. It has been widely used particularly in ophthalmology and other fields^3^ because of its non-invasive nature and high resolution. Fourier-domain retinal OCT^4,5^ is able to image biological tissues at a cellular level, and up to the depth of 1 mm below the retinal surface with high image quality. Moreover, the image-acquisition speed of OCT systems has been enhanced along with the development of high-speed sensors and tunable lasers with MHz scanning rate, to facilitate real-time retinal imaging.

OCT depends fundamentally on the coherence of the light used in the imaging process, and hence the reflection of a laser beam from a rough surface has a distinctive granular or mottle appearance^6^. The dark and bright spots formed by the reflected beam have no obvious relationship with the surface texture, which are often seen as noise. The noise pattern will change if the surface moves slightly^7^. The visualization quality of retinal OCT is often degraded by noises from different sources such as limited light bandwidth, phase aberrations of propagating beam, the aperture of the detector and multiple scatters within the coherence length^8^.

In medical diagnosis and therapy, besides affecting the overall image clarity, image noise and low contrast of images will also affect the image segmentation of lesion areas, which is critical for medical diagnosis and treatment. A large amount of noise will also reduce the positioning accuracy of surgical instruments in intraoperative retinal OCT imaging. It is hence essential to enhance the medical image quality by removing noise from OCT images, and preserving image micro-structures such as edges at the same time. In current commercial retinal OCT machines, a common optical approach to improve the quality of OCT images is to conduct averaging through overlapping scans^9,10^ or incoherent averaging^11,12^, but it has two disadvantages: (1) image quality degradation due to eye movement, which often happens in a clinical settings, (2) extending the acquisition time so that it may take too long for OCT imaging.

In recent years, a lot of denoising algorithms for OCT images have been developed to improve the image quality, which can be broadly divided into three categories: deep-learning-based^13,14^, sparse-coding-based^15–18^ and filter-based^19–21^. Deep-learning-based algorithms often over-fit to training data and have poor generalization performance, e,g, if training and testing images are obtained from different imaging sources, the denoising results would degrade significantly. OCT images from different machines or different manufacturers exhibit very different noise characteristics, so it is challenging to develop a deep-learning-based algorithm to well remove the noise in OCT images.

Regarding sparse-coding-based algorithms^16,22^, Cheng et al.^16^ reconstructed each A-scan for noise reduction and preserved the contrast in 3D OCT based on low rank matrix completion using bilateral random projection. Algorithms using dictionary learning^17,18,23^ have also been applied in retinal OCT image denoising. The selected dictionary atoms indicating the low quality image correspond to the counterpart atoms for recovering the high-quality image. Regarding filter-based algorithms, block matching & 3D collaborative filtering (BM3D) and its extension BM4D^24^ have been deployed to utilize small structures to remove the image noise. They stacked similar 2D/3D patches together and jointly removed the noise in a 3D/4D transform domain. Denoising algorithms often suffer the drawback of producing images with artifacts or low edge contrast.

Among filter-based approaches, wavelet-based denoising algorithms have been applied on OCT images, demonstrating excellent ability in reducing speckle noise and preserving image sharpness^25–27^. But it is widely known that the tranditional wavelet methods do not perform well in dimensions larger than one^28^. Thus it can not denoise well the retinal OCT image in a 3D volume. To address such a limitation, shearlets form a set of well localized filters at various scales and directions, and shearlet transformation holds the advantages of edge preserving and directional sensitivity, which provide a better representation of edge information than wavelet or curvelet transform^29–31^. We thus propose to remove the noise in retinal OCT images in the shearlet domain.

Directly applying shearlet filters to retinal OCT images may not produce high-quality images in a clinical setting. The reasons behind are two-fold: (1) The shearlet transformation for denoising usually models the noise distribution as Gaussian, and the noise is assumed uncorrelated to the signal^29,30^. The noise in retinal OCT images actually is signal-dependent^32,33^ and its distribution fits better to a Poisson distribution^34,35^. Thus, the OCT noise needs to be transformed to a Gaussian distribution so that the subsequent shearlet transform can work optimally. (2) The image noise varies significantly as images are acquired from retinal OCT systems built by different manufacturers, following different image acquisition practice by different clinical personnels for different imaging objects^26^. Therefore, it is difficult to use one fixed threshold to recover the clean image from the noisy data in the shearlet domain under different noise conditions^36^. Some noise may still remain in the filtered images if the threshold is too small, or some detailed information may be lost if the threshold is too large. Both situations are problematic in medical image analysis.

To solve the aforementioned two problems, we propose an image denoising algorithm, adaptive 3D shearlet Image-filtering with Noise-redistribution (referred as adaptive-SIN). Before applying the shearlet transform, the OCT noise is transformed to fit better to Gaussian distribution using the proposed square-root transform. Then, the shearlet transform is applied to remove the OCT noise and preserve edges and other image details. Then, an adaptive thresholding scheme is applied to better filter the noise in various noise conditions. Finally, the inverse shearlet transform is used to reconstruct the filtered image. The proposed adaptive-SIN is compared against other state-of-the-art approaches on three benchmark datasets, and demonstrates superior performance in terms of 6 popular subjective evaluation criteria. In terms of visual inspection, the proposed adaptive-SIN generates high-quality retinal OCT images without significantly removing image details.

Our main contributions are summarized as follow: (1) The proposed square-root transformation could transform the Poisson noise in the OCT images to fit better to Gaussian distribution, so that the subsequent shearlet transform could better filter the noise. (2) To tackle the challenges that the OCT noise varies significantly under different noise conditions, an adaptive thresholding scheme is proposed, so that the shearlet transform could automatically adapt to the noise conditions and better remove the noise. (3) The proposed adaptive-SIN is systematically evaluated on three benchmark datasets, and demonstrates superior performance in terms of both objective visual inspection and subjective quantitative evaluation.

## Proposed adaptive-SIN algorithm

OCT images are prone to noise since heterodyne detection used in OCT imaging achieves a detection sensitivity that approaches the quantum limit of a single photon. The noise in retinal OCT images often masks image details and poses significant challenges. Retinal images are governed by anisotropic structure^37^. The theory of shearlets empowers optimal encoding of several classes of multivariate data through the ability of sparsely representing anisotropic features. The retinal OCT data are often composed by a volume containing hundreds of B scans. As the shearlets use shearing to control directional selectivity, allowing shearlet system to be derived from a single or finite set of generators, so that it holds highly optimal approximation characteristics in all dimensions. Thus we propose our denoising algorithm based on the shearlet transformation. Nonetheless, the shearlet transformation may overlook two important aspects. Firstly, the shearlet transform for noise removal often assumes that the noise follows Gaussian distribution^29^, similarly as many other noise-removal algorithms do^38^. On the contrary, the noise in retina OCT images follows closer to a Poisson distribution than a Gaussian distribution, as evidenced in the papers^32–35^. We hence propose the square-root transform to re-distribute the OCT noise to fit better to Gaussian distribution, so that the subsequent shearlet transform could optimally remove the OCT noise. The second issue is that a fixed threshold is often used in the shearlet domain to estimate the clean signal from the noisy data. However, the nature of OCT noise varies significantly due to many factors such as different imaging objects, different imaging practice and different manufacturers. It is hence challenging to separate the signal from the noise using one fixed threshold in the shearlet domain. We hence propose an adaptive thresholding scheme for shearlet transform to better remove the noise and obtain OCT images of higher quality. All methods are performed in accordance with the Declaration of Helsinki, and approval has been obtained from all the subjects.

**Figure 1:**
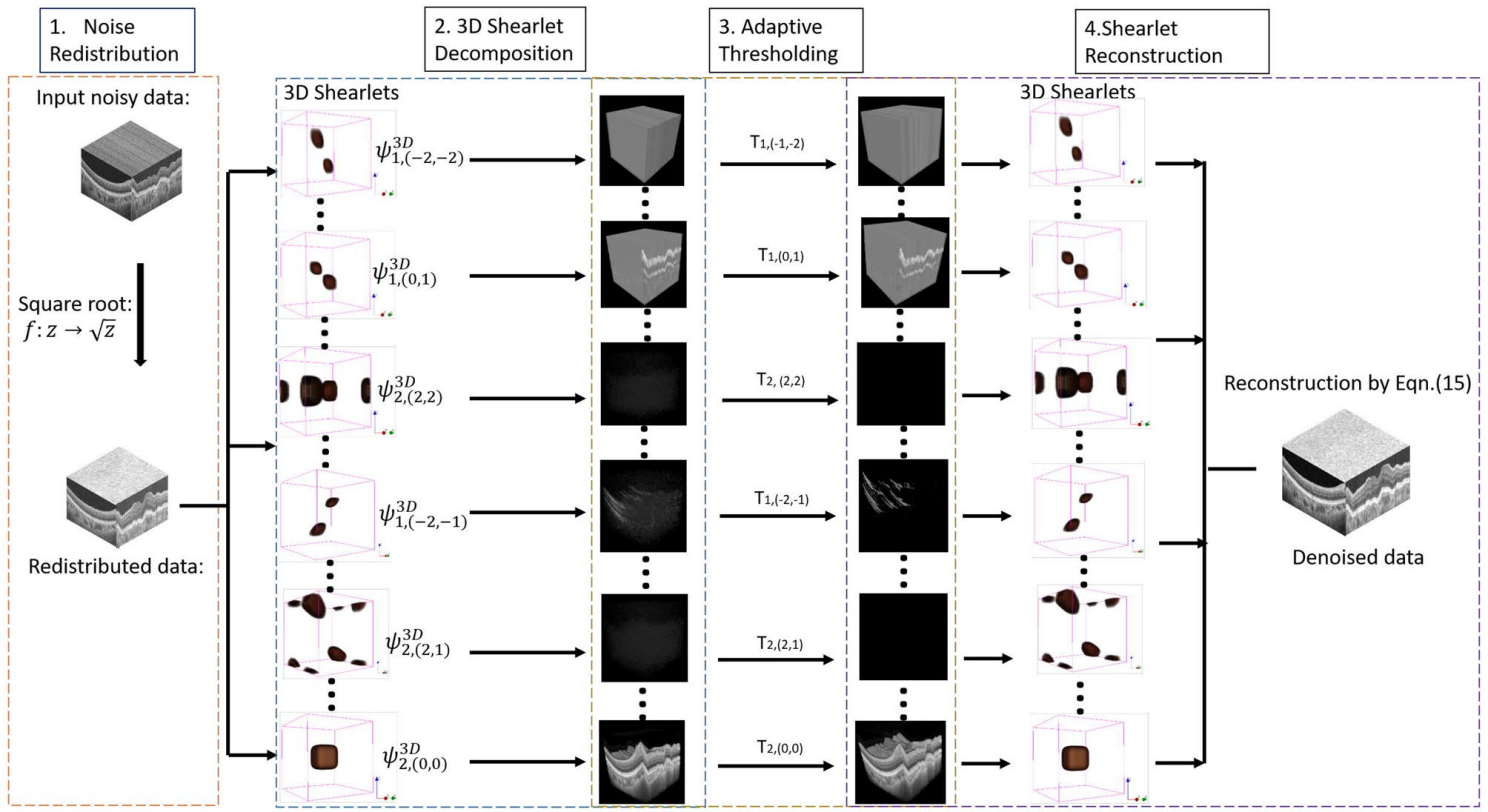
The flowchart of our proposed adaptive-SIN algorithm. In the algorithm, the noise in OCT images are redistributed, and then the redistributed images are decomposed by 3D shearlet transformation, adaptively thresholded to remove the noise and finally reconstructed to obtain the high-quality OCT images.

The proposed adaptive-SIN algorithm consists of four steps, as shown in Fig. 1. Firstly, we propose a square-root transform to redistribute the OCT noise to a Gaussian distribution. In the second step, the OCT images are decomposed using 3D shearlet domain so that edges can be better preserved in the shearlet domain. Thirdly, an adaptive thresholding scheme is proposed in the 3D shearlet transform so that the OCT noise can be better removed. Lastly, the inverse shearlet transform is applied to reconstruct the filtered images.

### Noise redistribution

The OCT noise follows closer to a Poisson distribution, as shown in the researches^34,35^. We hence need to redistribute it into a Gaussian distribution so that the subsequent shearlet transform works optimally. Formally, denote $z_{i}, i=1,2,...,N$ as the observed pixel values acquired from an OCT imaging device, which is modeled as an independent Poisson random variable, and its mean $y_{i}\ge 0$ is the underlying intensity value to be estimated. The discrete Poisson probability of $z_{i}$ is

1 $$\begin{aligned} P(z_{i}\mid y_{i}) = \frac{ y_{i}^{z_{i}}e^{-y_{i}}}{z_{i}!}, \end{aligned}$$

where the parameter $y_{i}$ is the mean of the Poisson variable $z_{i}$, and its variance is also $y_{i}$.

Then, Poisson noise can be officially defined as: $\eta _{i} = z_{i} - E\left\{ z_{i}\mid y_{i}\right\}$. The noise deviates largely from the Gaussian distribution. We could easily show that the mean noise is $E\left\{ \eta _{i}\mid y_{i}\right\} =0$ and its variance is $var\left\{ \eta _{i}\mid y_{i}\right\} = var\left\{ z_{i}\mid y_{i}\right\} = y_{i}.$

**Figure 2:**
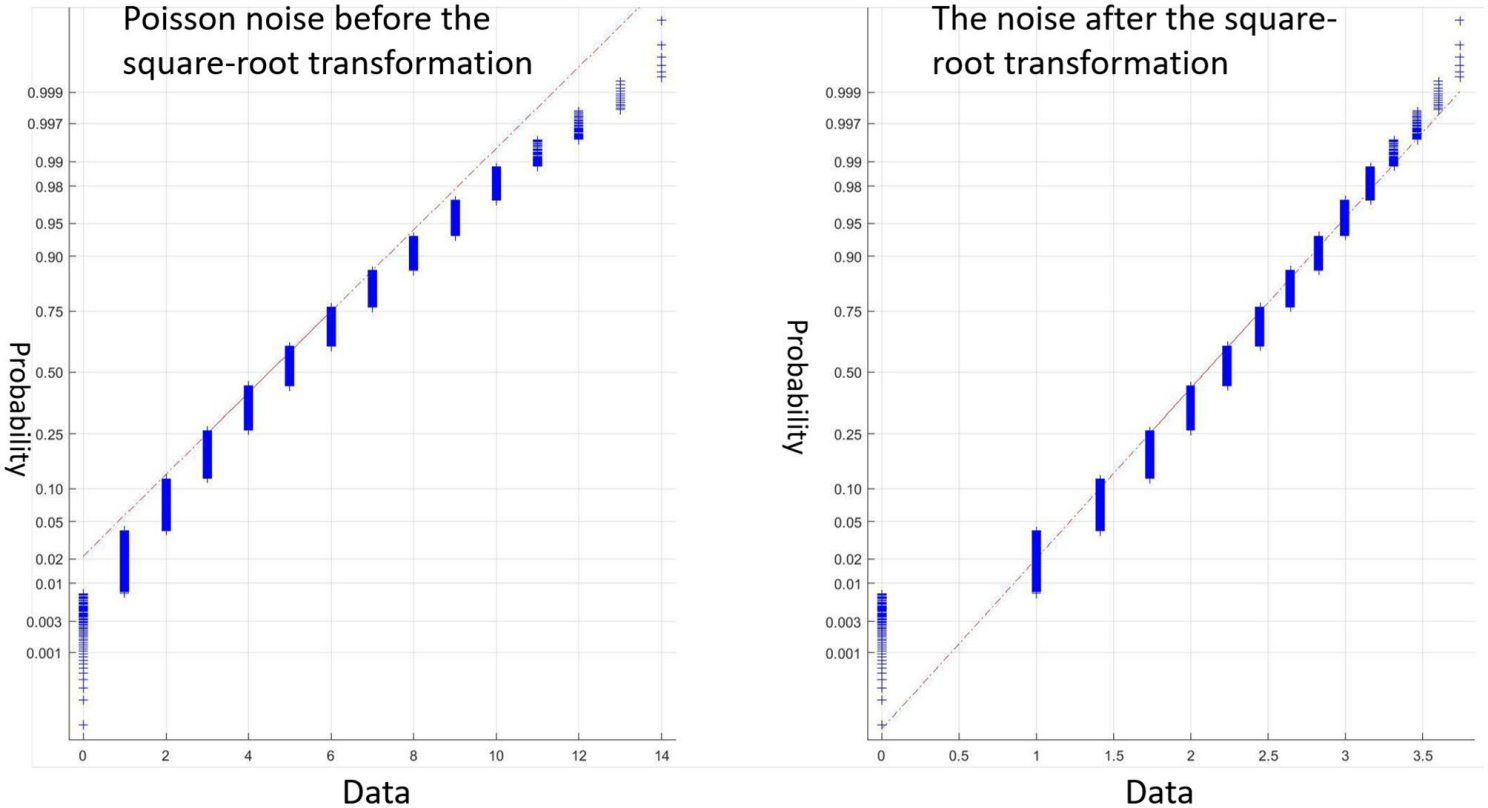
The normal probability plots for the Poisson noise and the transformed noise after square-root transform. It can be seen that after the transform, the data fit better to a Gaussian disbtribution.

In literature, many transforms have been proposed to transform data into Gaussian distribution^39–43^. For example, Anscombe transformation^39^ has been used to transform a Poisson distribution to a Gaussian distribution and Chi-square transform^43^ or log transform^44^ has been used to transform histogram-like features to a Gaussian distribution. In this paper, we propose a simple yet effective way, square-root transformation, to transform the noise in retinal OCT images from a Poisson distribution to a Gaussian distribution, as shown in the following equation:

2 $$\begin{aligned} f:z \rightarrow \sqrt{z}, \end{aligned}$$

where *z* is the OCT noisy data and *f* is the square-root transform. A rigorous proof to show that the transformed data fit better to a Gaussian distribution is tedious and beyonds the scope of this paper. Here, we use a synthetic example for illustration. We randomly select 10000 data samples from a Poisson distribution with a mean value of 5, and transform them using the proposed square-root transformation defined in Eqn. (2). We use the normal probability plot to assess the fitness to Gaussian, as shown in Fig. 2.

It can be seen that the transformed data indeed fit better to a Gaussian distribution, i.e. the transformed data fit closer to the red straight line that represents the ideal Gaussian distribution. After the transformation, the OCT noise is approximately Gaussianly distributed, so that the subsequent 3D shearlet transformation could optimally remove the noise.

### 3D shearlet decomposition

The shearlet transformation^31^ exhibits highly directional sensitivity and optimal approximation characteristics, which makes it suitable to remove noise from images. It often assumes that the noise in a given image is Gaussianly distributed. After the square-root transform, we assume an additive noise model as follows:

3 $$\begin{aligned} z= c + \varepsilon , \end{aligned}$$

where *c* is the underlying noiseless image and $\varepsilon$ is the redistributed Gaussian white noise with zero mean and standard deviation $\sigma$, i.e. $\varepsilon \in N(0,\sigma ^2)$. Our adaptive-SIN aims to recover the image from the noisy data *z* by computing an approximation of the noiseless image *c* through the proposed adaptive thresholding scheme in the subbands of the shearlet decomposition.

**Figure 3:**
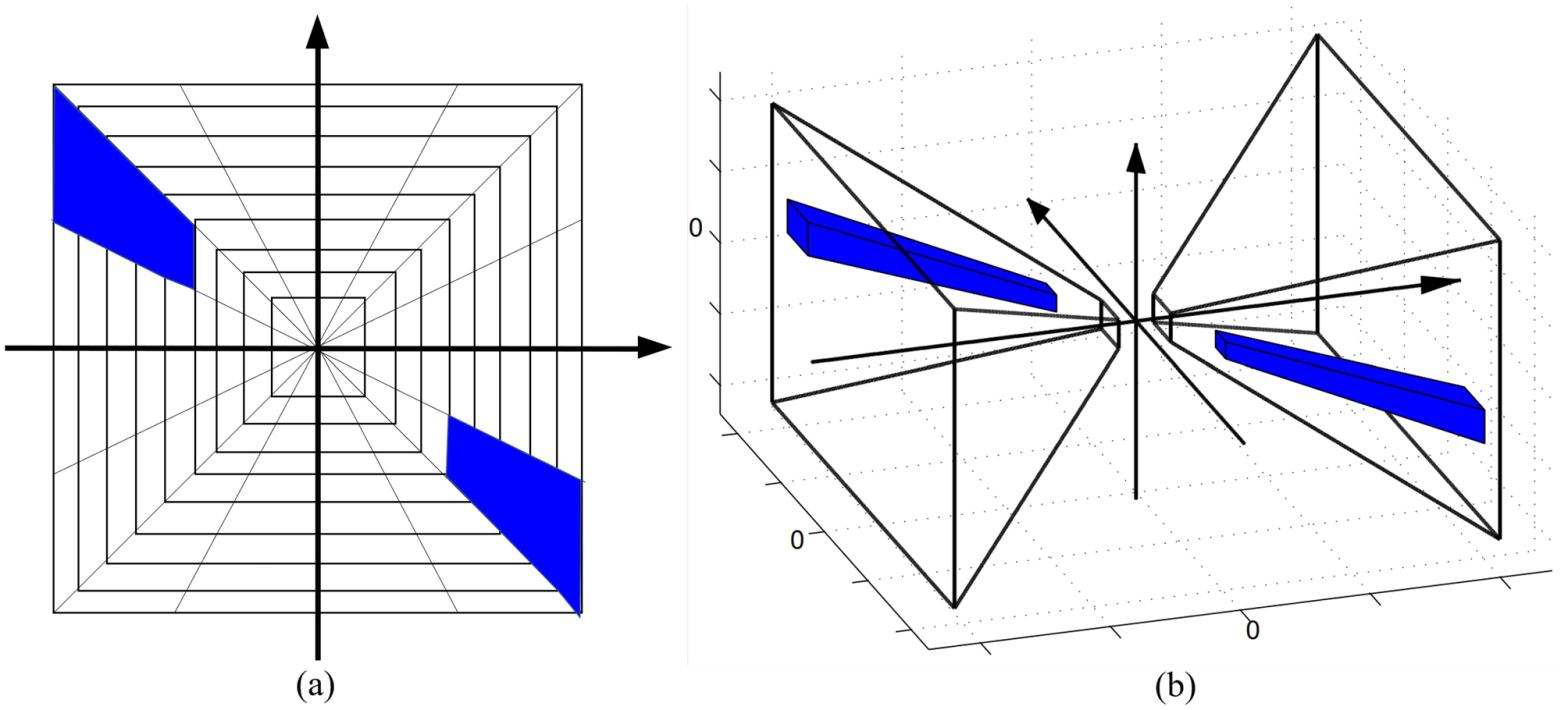
The difference between the 2D and 3D shearlets. (**a**) A representative shearlet function for 2D transformation. (**b**) A representative shearlet function for 3D transformation.

The shearlet transformation provides a way to decompose an image using functions ranging not only at various scales and locations, but also according to various orthogonal transformations controlled by shearing matrices. The shearlet transform for *z* is defined as:

4 $$\begin{aligned} \mathbb {ST}(a,k,t) = \langle z, \psi _{a,k,t} \rangle , \end{aligned}$$

where $\psi _{a,s,t}$ are generating functions defined as $\psi _{a,s,t}(z) = \left| M_{a,k} \right| ^{-1/2}\psi (M_{a,k}^{-1}z-t)$. $M_{a,k}$ is a dot product of a shearing matrix $$\begin{pmatrix} 1 &{}k \\ 0 &{}1 \end{pmatrix}$$and an anisotropic dilation matrix $$\begin{pmatrix} a &{}0 \\ 0 &{}\sqrt{a} \end{pmatrix}$$. *a* denotes the scale, *k* denotes the shear and $t \in {\mathbb {R}}^2$ denotes the translation. The 2D shearlets are derived by 1D shearing function and 1D scaling function applied in horizontal and vertical directions, as shown in Fig. 3a.

A retinal OCT volume generally contains hundreds of frames. The inter-frame information, such as the continuity of vessels is important for disease diagnosis. The 2D shearlet transform only adopts $2*2$ shearing matrices to capture the spatial information in a 2D space, which makes it unsuitable to capture the inter-frame information in a 3D scenario for the OCT retinal volume. To address this problem, we propose to adopt 3D shearlet transformation^45^ for the OCT volume denoising, as shown in Fig. 3b. The 3D shearlet transformation effectively extracts the information in a 3D space, which is helpful to preserve the continuity between frames.

**Figure 4:**
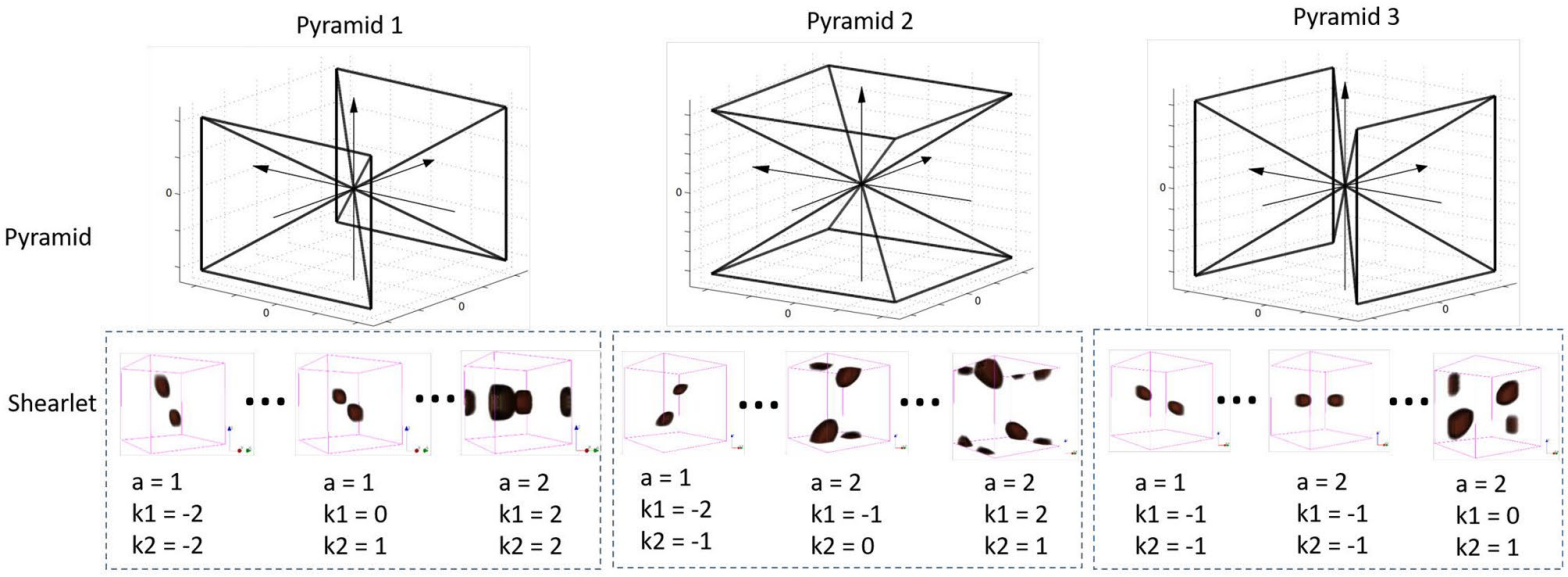
The pyramids for 3D shearlet decomposition, and some shearlet examples with corresponding parameters of scales and shears are shown in the figure. The parameter *a* denotes the scale of the corresponding shearlet filter while the shear parameter $k_1, k_2$ controls the orientation.

The three pyramids and some examples of the 3D shearlets are shown in Fig. 4. As shown in the first row of Fig. 4, the volume is partitioned into three pyramids to obtain the coarse shearlet features. Then the 3D shearlets related to the pyramidal regions are defined as the collections:

5 $$\begin{aligned} \left\{ \psi _{a,k,t}^{3D}:a\geqslant 0,-2^a\leqslant k_1, k_2 \leqslant 2^a, t\in {\mathbb {R}}^3 \right\} \end{aligned}$$

where $d=1,2,3$, $k=(k_1,k_2)\in {\mathbb {R}}^2$. The anisotropic dilation matrices for different pyramids are given by $$A_{(1)}=\begin{pmatrix} a &{} 0 &{} 0\\ 0 &{} {{a^{1/2}}} &{} 0\\ 0 &{} 0 &{} {{a^{1/2}}} \end{pmatrix}, A_{(2)}=\begin{pmatrix} {{a^{1/2}}} &{} 0 &{} 0\\ 0 &{} a &{} 0\\ 0 &{} 0 &{} {{a^{1/2}}} \end{pmatrix},A_{(3)}=\begin{pmatrix} {{a^{1/2}}} &{} 0 &{} 0\\ 0 &{} {{a^{1/2}}} &{} 0\\ 0 &{} 0 &{} a \end{pmatrix}$$. The shearings are related to a parameter $k=(k_1,k_2) \in {\mathbb {R}}^2$ in the shape of $${S_k}_{(1)} = \left[ {\begin{array}{*{20}{c}} 1&{}k_1&{}k_2\\ 0&{}1&{}0\\ 0&{}0&{}1 \end{array}} \right]$$, $${S_k}_{(2)}= \left[ {\begin{array}{*{20}{c}} 1&{}0&{}0\\ k_1&{}1&{}k_2\\ 0&{}0&{}1 \end{array}} \right]$$, $${S_k}_{(3)}= \left[ {\begin{array}{*{20}{c}} 1&{}0&{}0\\ 0&{}1&{}0\\ k_1&{}k_2&{}1 \end{array}} \right]$$. Thus the image decomposition based on the 3D shearlet transformation can be defined as:

6 $$\begin{aligned} \mathbb {ST}^{3D}(a,k,t) = \langle z,\psi _{a,k,t}^{3D} \rangle . \end{aligned}$$

In every pyramid, the transformation produces a series of shearlets by anisotropic scaling and shearing operators, which produce fine shearlet features. Those fine shearlet features shown in Fig. 4 could well capture the image micro-structures residing in different scales and different orientations. The shearlet filters are elongated with a larger scaling parameters, and those elongated shearlets could better capturhttps://www.overleaf.com/projecte image micro-structures such as edges than the isotropic scaling used in wavelets.

### Adaptive thresholding

Previously the noise variance in OCT signal is often assumed to be the same across images and the same threshold is applied to all the images^30,36^. However, the noise in OCT images has different characteristics for different manufacturers, different image acquisition practices and imaging objects. Therefore, we propose an adaptive thresholding scheme to take care of different noise characteristics in order to remove the noise more effectively.

As defined in the previous subsection, the noiseless component is denoted as *c*, the corrupted observation is denoted as *z*, and the noise component is denoted as $\varepsilon \in N(0,\sigma )$. To estimate the noiseless part, the standard maximum a posteriori (MAP) estimator^46^, maximum likelihood estimation (MLE)^47^ or minimum mean-squared error (MMSE)^48^ is often used to predict ${\hat{c}}$ given *z*. Take MAP as an example,

7 $$\begin{aligned} {\widehat{c}}(z) = \mathop {\arg \max }_{c} p(c|z), \end{aligned}$$

where *p*(*c*|*z*) is the posterior probability. In this solution, the noise is often assumed to follow the Gaussian distribution and its standard derivation $\sigma$ is assumed to be known in advance, so that the energy of the noiseless component can be estimated^49^, e.g.,

8 $$\begin{aligned} \sigma _c^2 = \sigma ^2_z-\sigma ^2, \end{aligned}$$

where $\sigma _z$ and $\sigma _c$ are the standard derivation of the noisy observation and the noise-free part, respectively. In this paper, we predict the noise variance from noisy shearlet coefficients^50^ as follows:

9 $$\begin{aligned} (\sigma _i)^2 = \frac{median(|z_i|)}{0.6745}, \end{aligned}$$

where $z_i$ is the $i_{th}$ shearlet, $\sigma _i$ is the noise estimation of the $i_{th}$ shearlet.

As the 3D shearlet filtering treats the input images as a volume, we apply the following equation to produce an estimation of the volume noise,

10 $$\begin{aligned} \sigma _{j} = \sqrt{\sum _i^{N} \sigma ^2_{i,j}}, \end{aligned}$$

where *N* is the number of shearlets and *j* indicates the test volume number.

**Figure 5:**
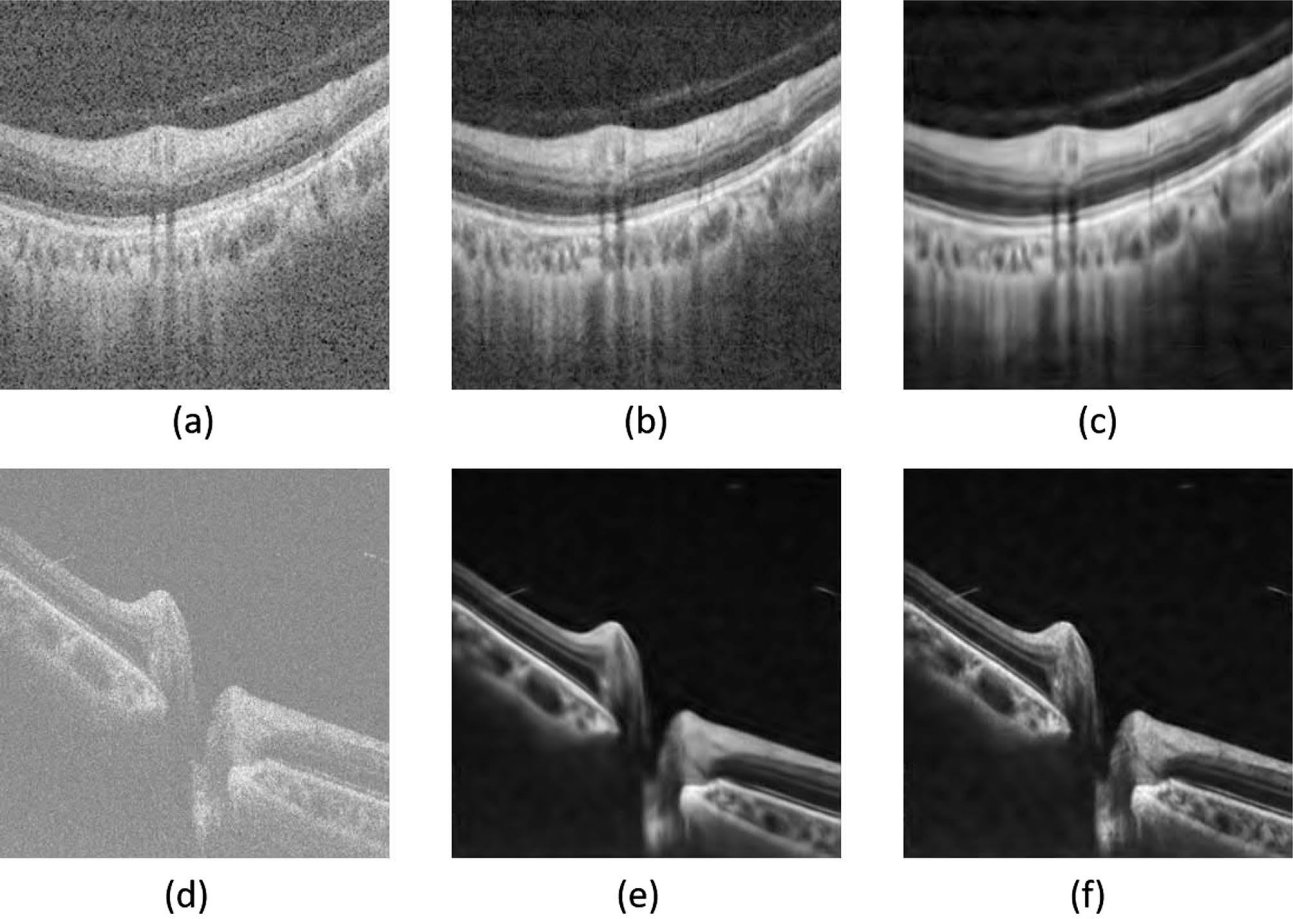
Image denoising with predefined or adaptive thresholds. (**a**,**d**) are noisy images, (**b**,**e**) are denoised by predefined thresholds, (**c**,**f**) are denoised by our adaptive thresholds.

Then for the adaptive-SIN, the threshold is adapted to the estimated noise as:

11 $$\begin{aligned} T_{a,k} = \frac{\sigma _j}{\sigma _{a,k}} \sigma _j , \end{aligned}$$

where $\sigma _{a,k}$ is the standard deviation of the shearlet coefficients in the (*a*, *k*) sub-band, indicating the signal intensity of (*a*, *k*) sub-band. The threshold is not only nearly optimal but also has an intuitive appeal. The normalized threshold $T_{a,k}$ is inversely proportional to sub-band standard deviation $\sigma _{a,k}$ and proportional to the noise standard deviation $\sigma _j$. When ${\sigma _j}/{\sigma _{a,k}} \ll 1$, the signal is much stronger than the noise, $T_{a,k}/{\sigma _j}$ is chosen to be small in order to preserve most of the signal and remove some of the noise, which can solve the problems of image details over-removing or some noise left caused by a predefined threshold. This adaptive threshold is then used to remove the noise. Below this threshold, the signal is recognized as the noise component and hence suppressed to 0. We show two examples in Fig. 5. The threshold is predefined as 30 as that in the previous paper^36^. There are lots of noise left in Fig. 5b using a predefined threshold, and the image in Fig. 5e is over-removed and some details are lost. Both of the above conditions are not beneficial to diagnose based on the images. The images in Fig. 5c,f are denoised by our adaptive thresholds, and the threshold values by adaptive thresholding are counted by the above equations as 48 and 24. They are denoised better than those using a predefined threshold. The detailed information near the disc part in Fig. 5 is apparently lost, which is not good for the doctors’ diagnosis or treatment plan.

### Shearlet reconstruction

Finally after adaptive thresholding an inverse shearlet transform provides a stable image reconstruction from the shearlet coefficients. The digital shearlet transform is a 3D convolution with shearlet filters, yielding a shift-invariant linear transform. Formally, the reconstruct image is obtained as

12 $$\begin{aligned} (\mathbb {ST}^{3D})^{-1}(a,k,t)=\langle s_{a,k,t},(\psi ^{3D})^{-1}_{a,k,t} \rangle , \end{aligned}$$

where $s_{a,k,t}$ is the shearlet coefficients and $(\phi ^{3D})^(-1)_{a,k,t}$is the inverse shearlet transform.

As shown in Fig. 1, the 3D denoised OCT images are reconstructed from the adaptively thresholding shearlet coeffients. In our experiments, a total of 99 shearlets are applied to capture OCT edges, curvilinear structures and texture.

## Experimental results

### Experimental settings

#### Datasets

In this paper, we evaluate the proposed algorithm on three datasets. All the experimental protocols were approved by the SUSTech ethics committee. The informed consent is obtained from all the subjects in the study.

##### Dataset of normal subject eyes

This dataset contains 20 3D retinal OCT volumes of eyes from 20 different subjects. For each normal subject, a 3D eye scan is obtained (containing 256 non-overlapping B-scan covering 6 mm × 6 mm × 2 mm region) using the Topcon ATLANTIS OCT machines with 3D-256 data acquisition mode. The Topcon OCT machine’s lateral resolution is 20 µm, and in-depth resolution is about 6 µm. Each B-scan has 512 A-scans, and each A-scan has 992 pixels. An objective measurement is important to evaluate the noise reduction performance. Although it is difficult to conduct overlapping scan to acquire high-quality 3D OCT due to the eye movement, we conduct line-scan at various locations and use the line-scan images as ground-truth for comparison. Similar ideas have been utilized in the literature^51,52^. In line-scan mode, the OCT machine repeatedly scans the same position for up to 96 times within seconds and the averaged B-scan image is expected to have much less noise effect and can be served as the ground-truth for noiseless OCT images. We conduct line-scan at three random locations for each 3D scan to have three line-scans per volume. The experimental evaluation is based on 60 different slices from 20 volumes.

##### Patients dataset from Topcon OCT machine

It contains 10 3D volumes for 10 patients’ eyes using Topcon ATLANTIS OCT machine. For each volume, a 3D scan is acquired containing 256 non-overlapping B-scan covering 6 mm × 6 mm × 2 mm region using the TOPCON ATLANTIS 3D-256 mode setting. Each B-scan has 512 A-scans, and each A-scan has 992 pixels. As those images are collected from hospital, we do not have the ground-truth noiseless images obtained using line-scan mode.

##### Patients dataset from Optovue OCT machine

It captures the OCT images of patients’ eyes using Optovue OCT machine, whose lateral resolution and in-depth resolution are 15 µm and 5 µm respectively. We collect 10 3D volumes from 5 patients of both eyes using Optovue OCT machine. For each eye, a 3D scan is obtained containing 304 non-overlapping B-scan covering 3 mm × 3 mm × 2 mm region using 3D mode setting. Each B-scan has 304 A-scans, and each A-scan has 640 pixels.

#### Compared methods

The proposed adaptive-SIN algorithm is compared with five state-of-the-art approaches, including A-scan reconstruction (ASR)^16^, the sparsity-based denoising (SBD)^17^, complex wavelet based K-SVD^18^, BM4D^24^, and a noise reduction method^53^ based on conditional generative adversarial network (cGAN). The proposed approach is also compared with three baseline approaches, including the original 2D Shearlet filter^29^ and 3D Shearlet filter^30^, 3D Shearlet denoising algorithm with noise-redistribution but constant denoising parameters (named ReShearlet)^54^. The constant denoising parameters are selected the same as those in the papers^36,54^ as 30. These three baseline approaches will show the gradual improvements of the proposed approach.

#### Parameter settings

All the experiments are conducted on the same computer using a dual core 2.20GHz CPU with 32 GB RAM. The number of shearlets is 99. The shearlet transformation is based on 3 dimensions. We use the recommended best parameters for all the above compared methods.

#### Evaluation metrics

The following six metrics are computed to evaluate the proposed adaptive-SIN algorithm.

##### Peak-signal-to-noise ratio (PSNR)

13 $$\begin{aligned} \begin{aligned} PSNR=10log_{10}\frac{I_{MAX}^2}{{\frac{1}{MN}}\sum {|I_F-I_G|^2}}, \end{aligned} \end{aligned}$$

where *M* and *N* are the number of rows and columns in the retinal OCT image, $I_{MAX}$ is the maximum pixel intensity, $I_{F}$ and $I_{G}$ denote the processed and ground truth OCT image respectively.

##### Mean square error (MSE)^55^

14 $$\begin{aligned} \begin{aligned} MSE=\frac{\sum (|I_{F}-I_{G}|)^2}{\sum I_{G}^2}. \end{aligned} \end{aligned}$$

##### Mean structure similarity index (MSSIM)^56^

15 $$\begin{aligned} \begin{aligned} MSSIM = \frac{1}{W}\sum {SSIM(x,y)}, \end{aligned} \end{aligned}$$

where the *SSIM* metric is calculated on various windows of an OCT image and *W* is the number of windows in the image. *x* and *y* denote the windows from $I_F$ and $I_G$. The measurement between *x* and *y* is given by:

16 $$\begin{aligned} \begin{aligned} SSIM(x,y)=\frac{(2\mu _{x}\mu _{y}+c_{1})(2\sigma _{xy}+c_{2})}{(\mu _{x}^2+\mu _{y}^2+c_{1})(\sigma _{x}^2+\sigma _{y}^2+c_{2})}, \end{aligned} \end{aligned}$$

where $\mu _{x}$ and $\mu _{y}$ are the average of *x* and *y*, $\sigma _{x}^2$ and $\sigma _{y}^2$ are the variance of *x* and *y*, respectively. $\sigma _{xy}$ is the covariance of *x* and *y*. $c_{1}=(0.01T)^2$ and $c_{2}=(0.03T)^2$ with *T* as the maximum value for data. The MSSIM is a measure of structural similarity between ground-truth and denoised OCT images, which is consistent with human perception. If MSSIM is closer to 1, it indicates a higher structural consistency between the denoised OCT B-scan and the ground-truth image.

##### Edge preservation index (EPI)^57^

Reflects the ability of preserving the image edges after denoising. To capture the edges of OCT images in the longitudinal direction, the EPI is defined as:

17 $$\begin{aligned} EPI=\frac{\sum _{i}\sum _{j}\left| I_{d}(i+1,j)-I_{d}(i,j) \right| }{\sum _{i}\sum _{j}\left| I_{n}(i+1,j)-I_{n}(i,j) \right| }, \end{aligned}$$

where, $I_d$ represents the denoised image and $I_n$ is the noisy image. *i* and *j* denote the *i*-th row and *j*-th column of the image. For EPI, 1 corresponds to prefect edge preservation.

##### Equivalent number of looks (ENL)

Indicates the smoothness in a homogeneous region. A higher ENL value indicates that the noise is better reduced from the homogeneous regions. The average ENL over *N* Region of Interests (ROIs) is calculated by:

18 $$\begin{aligned} ENL=\frac{1}{N}\sum _{r=1}^N\frac{\mu _{r}^2}{\sigma _{r}^2}, \end{aligned}$$

where $\mu _r$ and $\sigma ^2 _r$ are the mean and variance of the *r*-th ROI, respectively.

##### Contrast to noise ratio (CNR)

Is a measure of the contrast between a feature in ROI and the noisy background. The CNR over *r*-th ROI is defined as:

19 $$\begin{aligned} CNR_r = \frac{\left| \mu _{r} - \mu _{b} \right| }{\sqrt{0.5(\sigma _r^2+\sigma _b^2)}}, \end{aligned}$$

where $\mu _r$ and $\sigma _r^2$ denote the mean and variance of the $r-th$ ROI. $\mu _b$ and $\sigma _b^2$ denote the mean and variance of the background reference region. The value of CNR is significantly dependent on the features of the selected region, resulting into a large standard deviation across ROIs on different sample images. To consider multiple ROIs, the average CNR over N ROIs is computed ($N=5$ in the experiments). The above ROIs are randomly cropped from the retinal layer parts. The background reference regions are randomly cropped from the image background parts.

In the experiments, the aforementioned 6 metrics are used to evaluate the proposed approach on normal subject eye database. Only the last 3 metrics are applied on two image databases of patients’ eyes from different OCT machines, where no ground-truth is available. The experimental results are averaged on all the volumes in each database.

### Ablation study

To illustrate the performance improvement of each component of the proposed algorithm, the ablation study is conducted here based on the dataset of normal subject eyes. The proposed algorithm is compared with 2D shearlet, 3D shearlet and ReShearlet algorithms. We aim to evaluate the performance improvement from 2D shearlet to 3D shearlet, the performance gain brought by redistribution (i.e. ReShearlet over 3D shearlet) and the performance gain by adaptive thresholding (i.e. the proposed approach over ReShearlet). All the above six evaluation metrics are reported, as shown in Table 1. 3D shearlet outperforms 2D shearlets in terms of all 6 criteria significantly, which means that the 3D shearlets considering the inter-frame information could better remove the noise. The ReShearlet is 3D shearlet transformation based on data redistributed. The comparison between ReShearlet and 3D Shearlet shows that the noise redistribution improves the image quality. The comparison between the proposed approach and Reshearlet shows that in terms of 6 criteria (except the ENL a little lower than that by ReShearlet) the adaptive thresholding could better remove the image noise, because of the varying nature of OCT noise and the adaptive noise-removal mechanism in the proposed approach.

**Table 1:** Ablation experimental results based on PSNR, MSSIM, MSE, EPI, ENL and CNR. The bold numbers mean the best result compared with other algorithms.

	PSNR	MSSIM	MSE	EPI	ENL	CNR
2D Shearlet^29^	17.24	0.62	0.20	0.14	7.59	12.89
3D Shearlet^30^	20.43	0.70	0.13	0.44	19.42	16.42
ReShearlet^54^	21.87	0.73	0.07	0.48	**20.92**	16.39
Proposed	**22.51**	**0.78**	**0.06**	**0.88**	19.46	**16.43**

**Figure 6:**
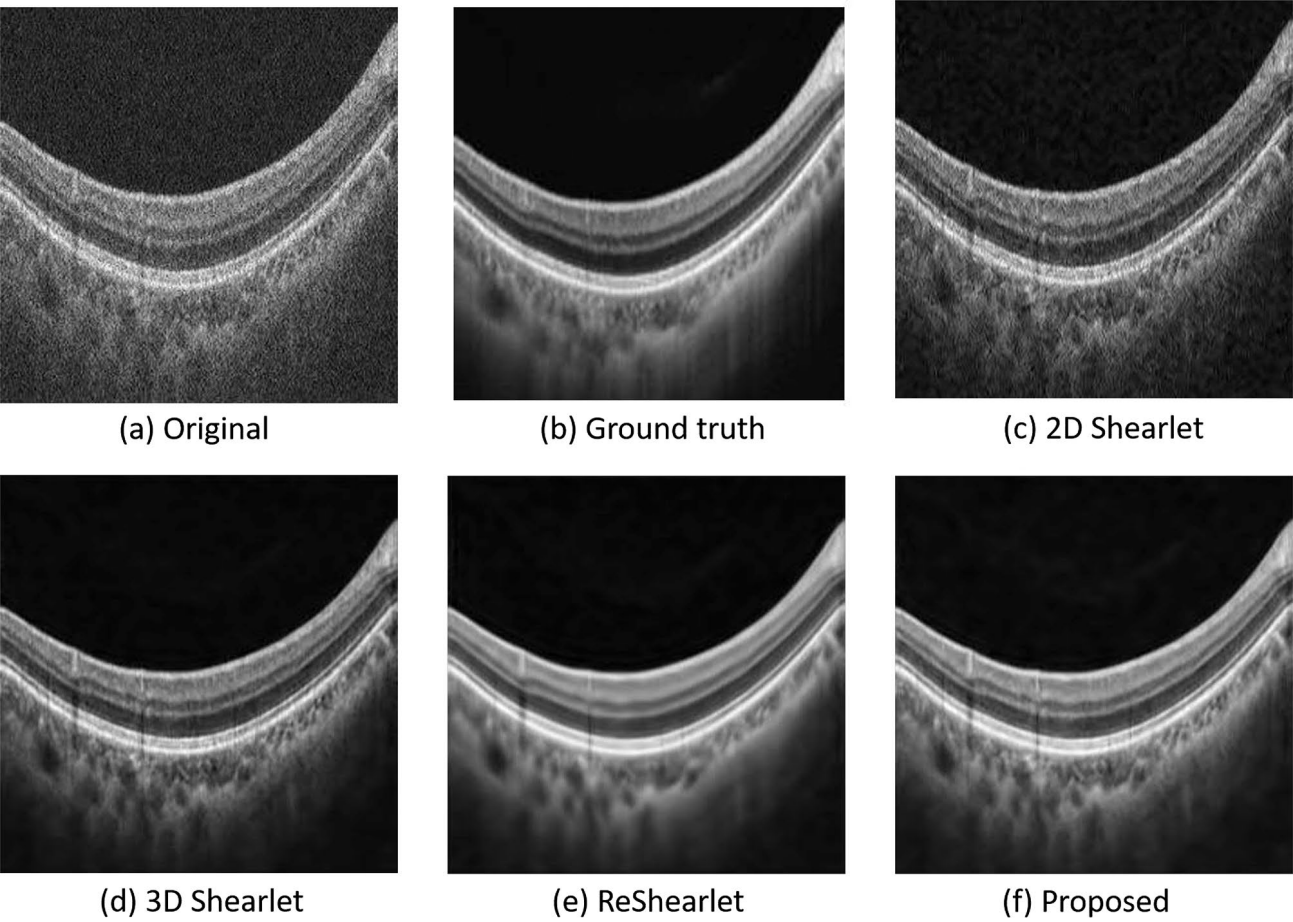
Normal sample images by our adaptive-SIN and other shearlet-related algorithms for the ablation study. (**a**) the original noisy image, (**b**) the ground-truth denoised image captured by multiple repeated scanning, (**c**)–(**e**) are the results by 2D shearlet, 3D shearlet and ReShearlet, (**f**) the result by the proposed algorithm.

To further illustrate the results, Fig. 6 shows the visual comparison between the proposed adaptive-SIN method and other shearlet-related methods. The denoised image by the original 2D shearlet remains noisy. The 3D Shearlet could remove more noise, because it adopts the inter-frame information, but some noise still remains. Figure 6e by ReShearlet loses some texture information, which is important for medical diagnosis and therapy. The proposed algorithm produces the image shown in Fig. 6f with detailed edge information, showing that the adaptiveness mechanism and noise redistribution are useful for the retinal OCT image denoising.

### Evaluation based on normal subject eyes

Table 2 shows the comparison to other 5 methods in terms of the six evaluation metrics. We can see that the proposed adaptive-SIN algorithm achieves the highest PSNR and MSSIM, as well as the lowest MSE. These clearly demonstrate the superiority of our adaptive-SIN algorithm in retinal OCT image denoising. The EPI, ENL and CNR of the proposed adaptive-SIN are highest in Table 2, which demonstrates the ability of edge preservation.

**Table 2:** Experiment results of our adaptive-SIN and other state-of-the-art algorithms are evaluated based on PSNR, MSSIM, MSE, EPI, ENL and CNR. The bold numbers mean the best result compared with other algorithms.

	PSNR	MSSIM	MSE	EPI	ENL	CNR
ASR^16^	17.17	0.45	0.08	0.18	4.68	13.21
SBD^17^	13.09	0.14	0.27	0.77	3.27	8.31
kSVD^18^	20.51	0.19	0.06	0.14	13.34	14.13
BM4D^24^	16.95	0.60	0.20	0.58	9.87	12.33
cGAN^53^	19.78	0.41	0.31	0.16	6.31	10.61
Proposed	**22.51**	**0.78**	**0.057**	**0.88**	**19.46**	**16.43**

**Figure 7:**
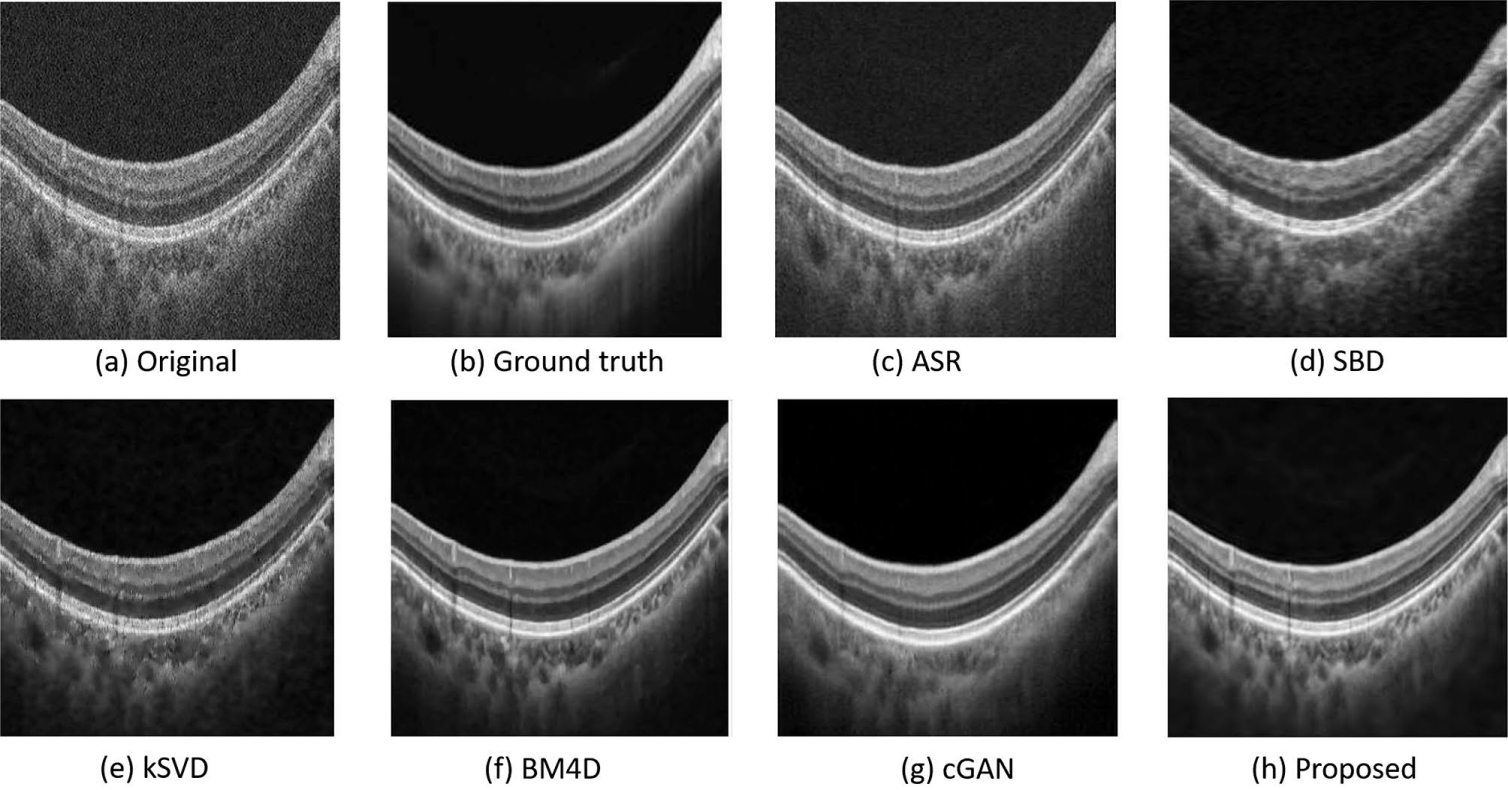
Normal sample images by our adaptive-SIN and other 8 algorithms for the performance evaluation. (**a**) the original image with noise, (**b**) the high-quality line-scan image as the reference to evaluate the denoised images, (**c**–**g**) results by traditional machine learning algorithms ASR, SBD, K-SVD BM4D, and cGAN respectively, (**h**) the result by the proposed algorithm.

Figure 7 shows the visual comparison between the proposed adaptive-SIN method and other 5 methods. The results in Fig. 7c–f show that the compared approaches may lead to artifacts or oversmooth in denoised images, which explains the lower ENL and CNR in Table 2. Figure 7g is an example of image noise by deep learning algorithm. It blurs the retinal OCT image and removes some textures in the OCT image, especially in the choroid layer. Figure 7h obtained by the proposed algorithm shows the highest edge contrast and least noise, and keeps the image details as much as possible. Those are consistent with the observation that the EPI, PSNR and MSSIM of our adaptive-SIN filtering algorithm in Table 2 are higher than other methods.

### Experiments based on patients images from topcon OCT machine

To evaluate the performance of the proposed adaptive-SIN algorithm on images with presence of disease lesions, comparison experiments are conducted on the patient image dataset from Topcon OCT machine. As those images are collected from hospital, we do not have the ground-truth noiseless images using line-scan mode. Thus the proposed algorithm is compared with others using three evaluation metrics including EPI, ENL and CNR. The results are averaged over 10 volumes of the dataset and summarized in Table 3. The proposed approach significantly outperforms all the compared approach in terms of all three evaluation criteria. The results show that the proposed algorithm produces the best edge preserving ability and the highest contrast.

**Table 3:** Experiment results of our adaptive-SIN and other 8 algorithms evaluated based on the metrics of EPI, ENL and CNR for the patients dataset captured from Topcon OCT machine. The bold numbers mean the best result compared with other algorithms.

	EPI	ENL	CNR
ASR^16^	0.10	2.17	4.72
SBD^17^	0.26	0.14	6.72
kSVD^18^	0.21	10.27	6.82
BM4D^24^	0.22	6.21	6.68
cGAN^53^	0.41	5.13	7.07
2D Shearlet	0.27	6.60	5.95
3d Shearlet	0.82	8.23	5.63
ReShearlet	0.85	10.93	5.98
Proposed	**0.87**	**11.35**	**8.67**

**Figure 8:**
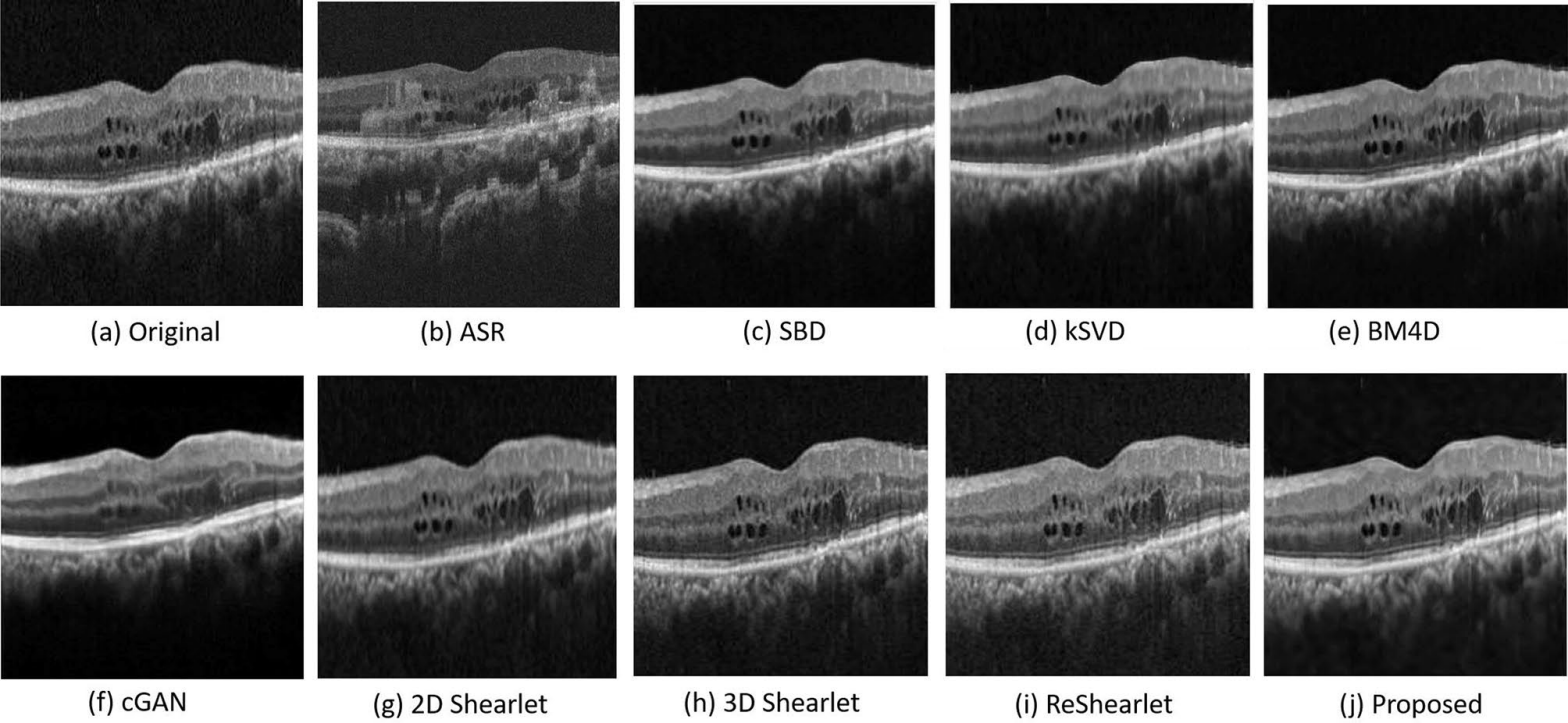
Sample OCT images of diseased eyes from Topcon OCT machine by our adaptive-SIN and other 8 algorithms for the performance evaluation. The proposed approach better removes the noise and preserves as many image micro-structures as possible.

Some examples of the denoised images are shown in Fig. 8. From Fig. 8, we can see that the denoised images by ASR have some artifacts in the choroid layer of the OCT. kSVD and 2D Shearlet may blur the images, and cGAN may remove some fine details in OCT images. We also find that the images filtered by 3D Shearlet and ReShearlet still contain some noise. The proposed adaptive-SIN algorithm produces high-quality image with fine details, which is consistent with the results shown in Table 3.

### Experiments based on patients images from Optovue OCT machine

To evaluate the proposed approach on OCT images by different machines, we test it on the images captured from Optovue OCT machine. We evaluate the image quality improvement using three evaluation metrics. The results are averaged over 10 volumes of the dataset, and summarized in Table 4. The highest EPI, ENL and CNR by the proposed algorithm shows that the proposed approach produces image with best quality, and could well preserve the image mirco-structures such as edges.

**Table 4:** Our adaptive-SIN and other 8 state-of-the-art algorithms evaluated based on the metrics of EPI, ENL and CNR for the patients dataset captured from Optovue OCT machine. The bold numbers mean the best result compared with other algorithms.

	EPI	ENL	CNR
ASR^16^	0.39	12.11	6.34
SBD^17^	0.19	7.49	5.73
kSVD^18^	0.20	10.80	6.25
BM4D^24^	0.17	5.91	6.30
cGAN^53^	0.29	3.27	5.08
2D Shearlet	0.19	7.59	7.45
3d Shearlet	0.49	11.88	6.56
ReShearlet	0.49	13.67	6.89
Proposed	**0.51**	**13.81**	**7.67**

**Figure 9:**
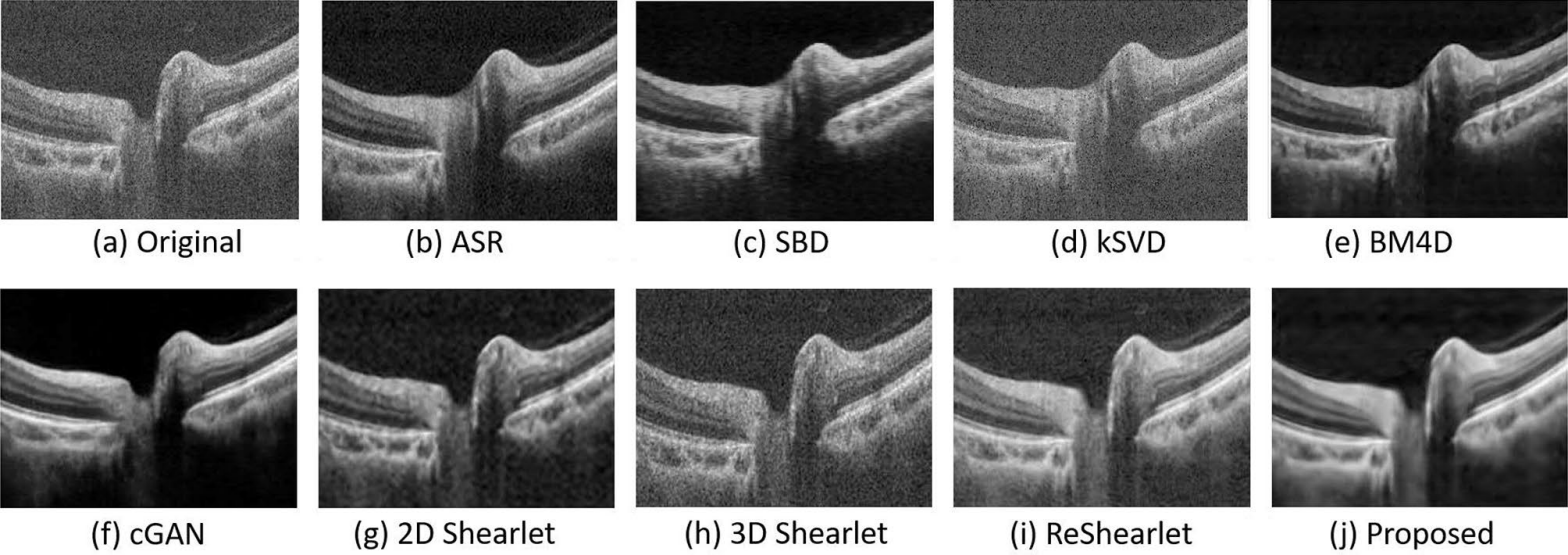
Sample OCT images from diseased eyes from Optovue machine by our adaptive-SIN and other 8 algorithms for the performance evaluation. The proposed approach better removes the noise and preserves the image micro-structures, and also exhibit better image constrast.

Some sample denoising results are shown in Fig. 9. SBD, BM4D and cGAN produce relatively clean backgrounds, but many fine image details are also removed as noise, which results in low EPI, ENL and CNR as shown in Table 4. On the other hand, methods such as ASR, KSVD, 2D Shearlet, 3D Shearlet and ReShearlet well preserve the image details, but a lot of noise are retained as well. Only our proposed adaptive-SIN well balances these two, i.e. preserving details as many as possible and removing noise as much as possible. Overall, the EPI, ENL and CNR are the highest for the proposed method.

## Discussion and conclusions

To tackle the challenge of reducing the OCT image noise of different natures, we propose an adaptive-SIN filtering algorithm. To facilitate the optimal noise-removal by the subsequent shearlet transform, the Poisson noise in the OCT images is transformed to the Gaussian noise by the proposed square-root transform. The 3D shearlet transform could well preserve the edge information in the images and other image fine details. The proposed adaptive thresholding scheme could well handle the diversified noise characteristics due to different manufactureers, different image acquisition protocols and different imaging objects. Extensive experiments have been conducted to verify the effectiveness of the proposed algorithm. Comparing with 8 other state-of-the-art denoising algorithms, the proposed adaptive-SIN achieves a superior performance on three benchmark datasets in terms of both objective quantitative assessments and subjective visual inspections. The denoised images can be used for OCT layer segmentation, which is the foundation for some retinal disease analysis and correlation analysis with diabetes, hypertension or some neurodegenerative diseases.
